# Application of MRI Post-processing in Presurgical Evaluation of Non-lesional Cingulate Epilepsy

**DOI:** 10.3389/fneur.2018.01013

**Published:** 2018-11-27

**Authors:** Shan Wang, Bo Jin, Thandar Aung, Masaya Katagiri, Stephen E. Jones, Balu Krishnan, Jorge A. Gonzalez-Martinez, Richard A. Prayson, Imad M. Najm, Andreas V. Alexopoulos, Shuang Wang, Meiping Ding, Zhong Irene Wang

**Affiliations:** ^1^Department of Neurology, Epilepsy Center, Second Affiliated Hospital, School of Medicine, Zhejiang University, Hangzhou, China; ^2^Epilepsy Center, Cleveland Clinic, Cleveland, OH, United States; ^3^Imaging Institute, Cleveland Clinic, Cleveland, OH, United States; ^4^Department of Neurosurgery, Cleveland Clinic, Cleveland, OH, United States; ^5^Department of Anatomic Pathology, Cleveland Clinic, Cleveland, OH, United States

**Keywords:** epilepsy, surgery, cingulate, MRI post-processing, non-lesional, focal cortical dysplasia

## Abstract

**Background and Purpose:** Surgical management of patients with cingulate epilepsy (CE) is highly challenging, especially when the MRI is non-lesional. We aimed to use a voxel-based MRI post-processing technique, implemented in a morphometric analysis program (MAP), to facilitate detection of subtle epileptogenic lesions in CE, thereby improving surgical evaluation of patients with CE with non-lesional MRI by visual inspection.

**Methods:** Included in this retrospective study were 9 patients with CE (6 with negative 3T MRI and 3 with subtly lesional 3T MRI) who underwent surgery and became seizure-free or had marked seizure improvement with at least 1-year follow-up. MRI post-processing was applied to pre-surgical T1-weighted volumetric sequence using MAP. The MAP finding was then coregistered and compared with other non-invasive imaging tests (FDG-PET, SPECT and MEG), intracranial EEG ictal onset, surgery location and histopathology.

**Results:** Single MAP+ abnormalities were found in 6 patients, including 3 patients with negative MRI, and 3 patients with subtly lesional MRI. Out of these 6 MAP+ patients, 4 patients became seizure-free after complete resection of the MAP+ abnormalities; 2 patients didn't become seizure-free following laser ablation that only partially overlapped with the MAP+ abnormalities. All MAP+ foci were concordant with intracranial EEG ictal onset (when performed). The localization value of FDG-PET, SPECT and MEG was limited in this cohort. FCD was identified in all patients' surgical pathology except for two cases of laser ablation with no tissue available.

**Conclusion:** MAP provided helpful information for identifying subtle epileptogenic abnormalities in patients with non-lesional cingulate epilepsy. MRI postprocessing should be considered to add to the presurgical evaluation test battery of non-lesional cingulate epilepsy.

## Introduction

Surgical management of patients with cingulate epilepsy (CE) is highly challenging, especially in the setting of negative MRI. Due to its mesial and deep location from the cerebral surface as well as the absence of unique ictal manifestations, scalp video-electroencephalography (EEG) may be misleading or non-localizable (1–4). The fast propagation of seizure activities originating from cingulate cortex (CC) within the limbic network (5), complicated functional connectivity between homotopic cingulate and sensorimotor cortex (3, 6), and diffuse bilaterally secondary synchrony of epileptiform discharges from cingulate lesions (2, 7) all contribute to the difficulty in localizing CE.

A confirmed MRI lesion can contribute directly to the identification of the epileptogenic zone (EZ) (8). When patients have no apparent lesions on the MRI, presurgical evaluation and surgical management can be particularly difficult, as seizure origin could be strongly influenced by the availability of collective expertise and experience in semiology, neurophysiological exploration, and functional imaging interpretation (4, 8). Previous studies with voxel-based MRI post-processing using a morphometric analysis program (MAP) (9) combined with visual MRI analysis indicated high sensitivity in the identification of subtle epileptic lesions (10–14); MAP+ findings was reported to provide valuable targets for invasive evaluation and resection (15). However, there was no study on the post-processing neuroimaging characteristics of CE with a normal pre-surgical MRI.

In the current study, we aimed to investigate the usefulness of voxel-based MRI post-processing to detect subtle abnormalities in CE with a negative pre-surgical MRI. In relation to the MAP findings, we examined the non-invasive electro-clinical characteristics and functional imaging findings in these patients. When possible, concordance with intracranial EEG finding was investigated.

## Materials and methods

### Patients

This retrospective study was approved by the institutional review board ethical guidelines of two hospitals: Cleveland Clinic Foundation (CCF) and the Second Affiliated Hospital of Zhejiang University (SAHZU). We reviewed a consecutive series of patients who had surgery at CCF from January 2008 to December 2016 and SAHZU from January 2013 to April 2017. The inclusion criteria were as follows: (1) intracranial-EEG (ICEEG) confirmed focal cingulate ictal onset during recorded habitual seizures, or resection of the cingulate cortex with/without adjacent cortex rendered the patient seizure-free or having marked seizure improvement with 1-year follow-up; (2) preoperative MRI and postoperative MRI/CT data were available; (3) preoperative MRI was considered as negative or suspicious of a subtle lesion during the multidisciplinary patient management conferences (PMC). Patients were excluded if they (1) had poor MRI quality; (2) had a definite lesion in the cingulate cortex on MRI; and (3) seizures recurred without a marked improvement after surgery. The vertical anterior/posterior commissure lines (VAV/VPC) were used as a landmark to divide the cingulate cortex into three parts: the anterior cingulate, located rostral to the VAC; the middle cingulate, located between VAC and VPC; and the posterior cingulate, located caudal to the VPC line (2).

### Presurgical evaluation

The surgical strategy was discussed based on pre-surgical evaluation including history, semiology, video scalp-EEG, MRI, FDG-PET, subtraction ictal SPECT co-registered with MRI (SISCOM), Magnetoencephalography (MEG) and ICEEG. Semiology based on history and video-EEG was evaluated with classifications developed by Lüders et al. (16). Results of pre-surgical evaluation tests were obtained from chart reviews of the patients' clinical files.

### Data acquisition and analyses

MRI post-processing was based with MAP07 within MATLAB 2015a (MathWorks, Natick, Massachusetts) and analyzed on a voxel basis (9) with comparison to a normal database consisting of 90 normal controls (17). Patients from CCF were scanned by 3.0-T MRI scanners (Trio or Skyra, SIEMENS, Erlangen, Germany) with T1-weighted Magnetization Prepared Rapid Acquisition with Gradient Echo images; patients from SAHZU were scanned with a 3.0-T MRI scanner (MR750, GE Healthcare) with a 3-dimensional (3D) T1-weighted Spoiled Gradient Recalled Echo sequence. Detailed parameters can be found elsewhere (18). The final outputs of MAP consisted of three feature maps, the *junction, extension*, and *thickness* maps. The *junction* map is sensitive to blurring of the gray-white matter junction; the *extension* map is sensitive to abnormal gyration and extension of gray matter into white matter; the *thickness* map is sensitive to abnormal cortical thickness (9). A blinded reviewer (Shan Wang) used a z-score threshold of 3 to identify candidate MAP+ regions in the junction file and then examined the suspect on extension (Z>6) and thickness (Z>4) files. The abnormality was reaffirmed by a neuroradiologist (SEJ), checking pre-operative MRI including T1-weighted, T2-weighted and FLAIR sequences to confirm MAP+ positive regions. In all MAP+ patients, we used SPM12 to co-register preoperative T1-weighted images, MAP files and postoperative MRI images in order to confirm whether the location of the MAP+ regions was included in the resection.

### Pathology and outcome

Surgical pathology, when available, was re-reviewed by dedicated neuropathologists from each hospital. The diagnosis and classification of FCD were performed according to the ILAE guidelines (19). Postoperative seizure outcomes were determined according to Engel's Classification (8). Engel Class 1 (seizure-free) and 2 (>90% reduction) were regarded as marked improvement of seizure frequency (2, 8).

## Results

### Patient population

Out of the 1,518 patients with a localized resection from the CCF surgical database, 21 patients had resection of the cingulate cortex; 17 of the 21 patients had strictly non-lesional MRI or had subtle cingulate abnormalities. Ten patients were further excluded because the invasive EEG onset was not merely limited to the cingulate region, or the resection included but extended beyond the cingulate cortex, or there was no marked seizure improvement after surgery. Out of the 240 patients with a localized resection from SAHZU, 7 patients had resection of the cingulate cortex; 3 patients had strictly non-lesional MRI. One patient was further excluded because the invasive EEG onset was not merely limited to the cingulate cortex. Therefore, a total of 9 patients were identified from the two Epilepsy Centers (7 from CCF), including 5 from anterior CE, 3 from middle CE and 1 from posterior CE. Six were females; the median age at surgery was 22 (range, 14.5–38) years; the median epilepsy duration was 60 (range, 13–173) months. Six patients with negative MRI underwent ICEEG monitoring, which confirmed cingulate focal ictal onset during their habitual seizures. Subtle CC abnormalities in three patients were identified during re-review at PMC and no ICEEG was recommended per PMC consensus for these 3 patients. Detailed clinical information, results of pre-surgical evaluation, pathology, and postsurgical seizure outcomes were summarized in Table 1.

**Table 1:** Summary of clinical profile, presurgical evaluation data, surgery, histopathology, and postoperative seizure outcome for the 9 included patients.

**Parameters/No**.	**P1**	**P2**	**P3**	**P4**	**P5**	**P6**	**P7**	**P8**	**P9**
Age range (Y)	26–30	11–15	16–20	11–15	31–35	21–25	41–45	41–45	21–25
Handedness	R	R	L	R	R	R	R	L	R
Epilepsy duration (m)	60	14	108	130	4	12	300	216	24
Seizure semiology	Dialeptic → hypomotor /gelastic sz	Complex motor sz	Aura (L hand somatosensory) → bil asymmetric tonic → L hand clonic sz	Aura (dizziness) → bil asymmetric tonic sz → R arm tonic sz	Aura (fear) → automotor/complex motor sz	Aura (fear) → Complex motor sz	Aura (L arm somatosensory) → automotor sz → sGTCS	Unclassified aura → hypermotor/complex motor sz	Complex motor sz
Ictal scalp EEG	L fronto-centro-parietal	L central	Non-localized	L frontal	R anterior mesial frontal	L hemisphere	R temporal	Non-localized	Bil Central
Invasive interictal EEG	L ACG/MCG/superior-middle frontal/precentral	L MCG	R MCG	NA	NA	NA	R PCG/hippocampus	L MCG	L ACG/mesial frontal
Invasive ictal EEG	L ACG/MCG	L MCG	R MCG	NA	NA	NA	R PCG	L MCG	L ACG
MAP	L ACG	L MCG	R MCG	L ACG	R ACG	L ACG	Neg	Neg	Neg
FDG-PET	L dorsomedial frontal	R parietal	Bil temporal; frontal operculum/insular, L>R	L fronto-temporal	Bil temporal/dorsal medial frontal	L temporal	R fronto-temporal	Bil parieto-temporal, L>R; left frontal	L anterior mesial frontal
SISCOM (injection time/seconds)	L fronto-parieto-occipital (14)	N/A	Bil dorsal posterior frontal; R occipital (16)	R dorsal parietal/mid-posterior lateral temporal (15)	NA	NA	NA	R fronto-temporal (12)	NA
MEG	L superior/middle frontal	L fronto-central (outside report)	Bil Central	NA	NA	NA	Bil temporal	Neg	NA
Pathology	Surgery 1: NA (laser) Surgery 2: Infarct/inflammation	NA (laser)	FCD IIb	FCD IIb	FCD IIb	FCD IIa	FCD Ib	FCD Ib	FCD IIa
Surgery	L ACG laser L ACG + superior frontal resection	L MCG laser	R MCG resection	L ACG resection	R ACG resection	L ACG resection	R PCG resection	L MCG resection	L ACG resection
Outcome	Surgery 1: II Surgery 2: IA	II	IA	IA	IA	IA	IB	IB	IA

*y, year; m, months; M, male; F, female; R, right; L, left; Bil, bilateral; sz, seizures; sGTCS, secondary generalized tonic clonic seizures; NA, not available; ACG, anterior cingulate gyrus; MCG, mid cingulate gyrus; PCG, posterior cingulate gyrus; Neg, negative*.

### Non-invasive pre-surgical evaluation

On scalp EEG, ictal onset lateralized to the ipsilateral hemisphere (fronto-centro-parietal = 1, central = 1, frontal = 2, temporal = 1, hemisphere = 1) in 6 of the 9 patients. FDG-PET was performed in all 9 patients; in only 2 patients, hypometabolism overlapped with (and also extended beyond) the CC (P1 and P9). Ictal SPECT was successfully obtained in 4 of the 9 patients (injection time: 12–16 s); the hyperperfusion areas contained the CC only in one patient (P1). MEG was performed in 5 of the 9 patients; positive findings were found in 4 patients, and only 2 of the 4 patients had MEG findings overlapping with the CC (P1 and P2, both loose clusters).

### MAP findings

**Figure 1:**
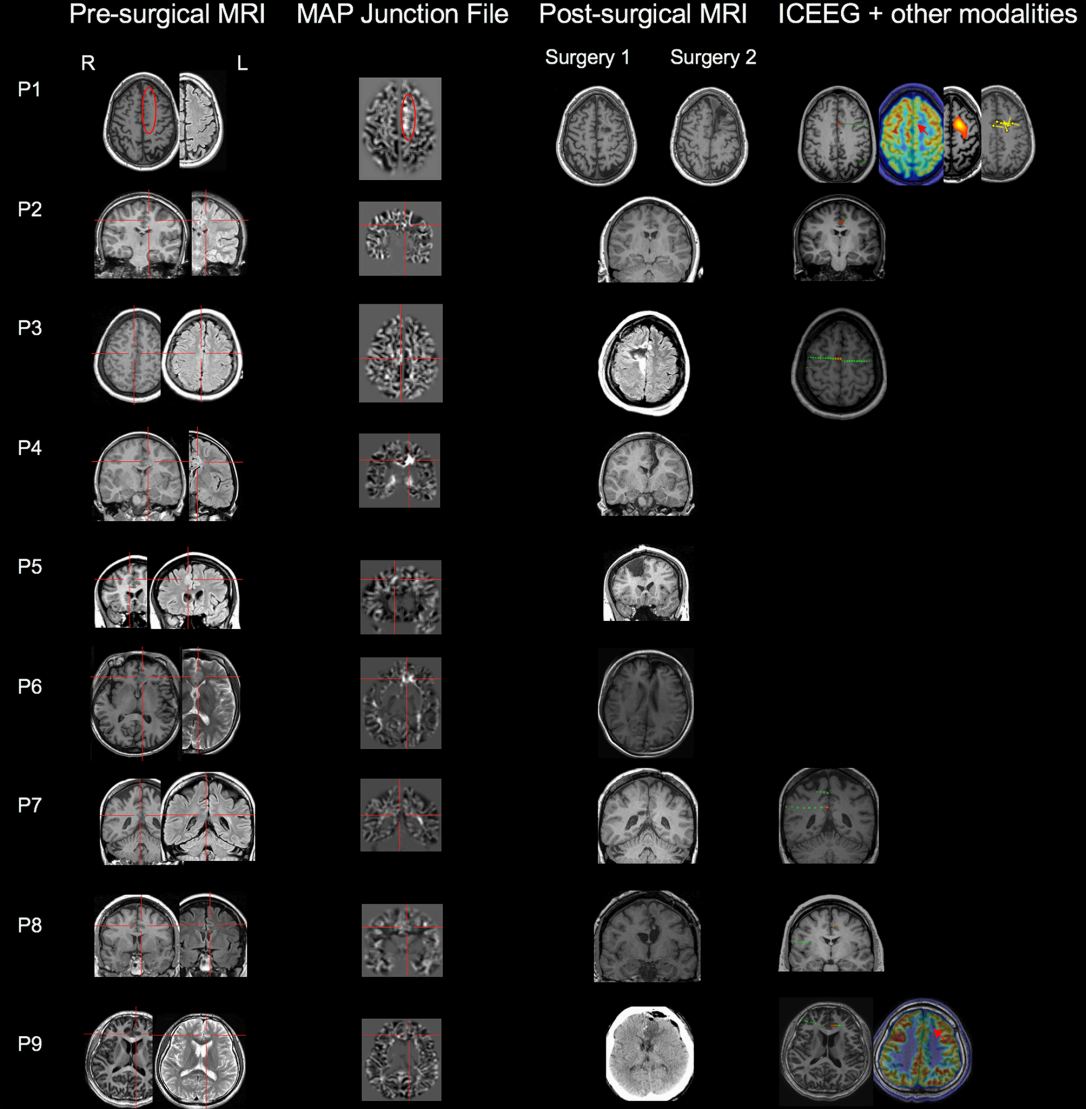
MAP findings illustrated for all 9 patients included in this study. Single MAP+ abnormalities were found in 6 patients (P1-P6), including P1-P3 who had negative 3T MRI by visual analyses, and P4-P6 who had subtly lesional 3T MRI by visual analyses. P7-P9 had negative MAP. First column: pre-surgical T1-weighted/FLAIR images; second column: co-registered MAP junction files; third column: post-surgical MRI indicating resection of the cingulate cortex. The red circle or cross hair shows the location of subtle abnormalities identified by MAP (in P7-P9, MAP was negative so the crosshair was set to the location of the ICEEG ictal onset). Fourth column shows ICEEG ictal onset and FDG-PET/SISCOM/MEG findings (if concordant). Red electrode contacts indicate ictal onset locations that are concordant with MAP+ findings. In P1, two surgeries were performed, 25 months apart. P1 had seizure recurrence at 15 months following laser ablation that partially overlapped with the MAP+ abnormality, and became seizure-free for 1 year after the second resection to clean up the resection margin, which included the entire MAP+ region.

The MAP findings are illustrated for all 9 patients in Figure 1. Single MAP+ abnormalities were found in 6 patients (P1-P6), including 3 of the 6 patients with negative MRI, and 3 patients with subtly lesional MRI. In P1-P3 who had negative MRI, MAP gray-white junction file pinpointed a subtle abnormality in the anterior or middle CC, which was found in retrospect to represent subtle blurring of gray-white matter junction in the original T1/FLAIR images, concordant with ICEEG (Figure 1). P4-P6 with subtly lesional MRI were all found to have abnormalities on MAP in the anterior CC; they did not have ICEEG as the subtle findings were identified during re-review at PMC. P7-P9 had negative MAP while their ICEEG showed focal ictal onset in the cingulate cortex. MAP extension or thickness files did not have additional yield; only in P4, a supra-threshold abnormality was seen on the extension file accompanying the junction file.

### Outcome, surgery, and pathology

Out of the 6 MAP+ patients, 4 patients (P3-P6) had the resection completely overlapping with the MAP+ region and became seizure-free; two patients (P1 and P2) didn't become seizure-free: P1 experienced seizure recurrence at 15 months following laser ablation that partially overlapped with the MAP+ abnormality, and became seizure-free for 1 year after the second resection to clean up the resection margin, which included the entire MAP+ region; in P2, who had marked improvement in seizure frequency and intensity (Class II), post-operative MRI indicated incomplete removal of the area corresponding to ICEEG and MAP. The 3 MAP-negative patients did become seizure-free (one Class Ia, two Class Ib) following resective surgery guided by ICEEG. Surgical pathology revealed FCD in 7 patients, including FCD type Ib (*n* = 2), type IIa (*n* = 2), and type IIb (*n* = 3). No specimen was sent to pathology examination in the two patients who had laser ablation.

## Discussion

Non-lesional cingulate epilepsy is a rare form of epilepsy (2). Our current study presents the largest series of patients with surgically confirmed non-lesional cingulate epilepsy, with utility of MRI postprocessing to help identify subtle structural abnormalities in this challenging cohort. We showed that voxel-based MRI postprocessing identified subtle epileptic abnormalities in the majority of patients, while the localization value of scalp EEG, PET, ictal SPECT, and MEG was relatively limited. This finding emphasizes the practical value of adding MRI post-processing into the presurgical evaluation workflow of MRI-negative cingulate epilepsy.

Surgical management of patients with CE is challenging, as CE exhibits significant heterogeneity in its manifestations due to different seizure propagation patterns (3). Animal and human studies have demonstrated that the anterior CC is bi-directionally connected to the prefrontal and premotor areas, and the posterior CC bi-directionally connected to the mesial temporal regions (1, 3, 20, 21). Moreover, epileptic discharges from the CC often present secondary bilateral synchronous epileptiform discharges, which increases the difficulty to precisely localize (22). Not surprisingly, scalp EEG was less helpful to localize EZ located in the CC because of its low spatial resolution and inability to detect deep focus (3). Complex epileptic networks and fast propagation of discharges from the CC could account for the relatively low yields of PET and SISCOM as reported in previous studies (1, 2, 12, 23). Wong et al. (24). demonstrated that rapid spread of epileptic activities could result in widespread hypometabolism, sometimes remote to the EZ. Diffuse regions of hyperperfusion might reflect the epileptic network which includes the epileptic focus as well as the propagation pathways away from the onset, further complicating the task of localization (25). Although MEG has theoretical advantages including high spatial and temporal resolution in identifying epileptic activities from deep structures compared to scalp EEG (26), its localization seemed to be limited for CC as shown in our study, perhaps due to the CC producing radially oriented sources difficult to be detected by MEG source localization.

In 50% (3 of 6) of the patients with CE and negative MRI in our series, abnormalities were identified using MAP; in all 3 patients with CE and subtly lesional MRI, abnormalities were identified using MAP; the overall detection rate was analogous to published series whose detection rate ranged between 43 and 50% in MRI-negative epilepsies (12, 13). FCD is the most common identifiable pathology among MRI-negative epilepsies, frequently presenting blurring of the gray-white matter junction (11). Therefore, it is expected that junction map was the most helpful feature map in the current study and previous studies (11–13). The majority (4 of 5) patients with FCD type II were successfully detected by MAP in our study, while neither case with FCD type I (P7-P8) was MAP+. Therefore, the type of the underlying pathology likely contributes to the negative MAP results. It's a considerable challenge to identify and demarcate FCD type I by current MRI techniques even in patients with confirmed histopathology (27), as FCD type I is typically not as well-characterized on the MRI with less prominent features. Our previous study looked at a group of 150 MRI-negative epilepsies which mostly consisted of FCD type I; not all patients with positive pathology of FCD type I were MAP+; additionally, 5 patients with FCD type I had seizure recurrence even though resection fully overlapped with their MAP+ regions, which suggests insufficient delineation of the full extent of the FCD type I using the current technique (13). Another point worth noting is that the T1-based MAP processing, as utilized in this study, would not be able to capture subtle FCDs with a strong T2 change but no T1 change. This could be another factor contributing to negative MAP results. In the face of a completely non-lesional MRI (visual-negative and MAP-negative), ICEEG is often mandatory to explore the epileptogenic zone.

Being seizure-free is the gold standard to identify epileptogenic characteristics of MAP+ changes (11, 12). In the current study, MAP+ findings were included in the surgical resection in 4 patients with seizure freedom, suggesting that these findings were true positive findings. The two patients who didn't become seizure-free both had laser ablation which partially overlapped with their MAP+ abnormalities; the less optimal seizure outcomes might be due to the incomplete removal of the epileptic structural abnormality. The type of surgery could also be contributive; although minimally invasive, laser ablation was reported to be less effective than conventional resective surgery in a prior study on 19 pediatric patients (28).

## Limitations

Patients studied here were a highly selected cohort and could not represent all patients with cingulate epilepsy. Using a combined dataset from two epilepsy centers, there might have been differences in the interpretation of presurgical evaluation tests and surgical decision. These limitations should be considered when interpreting results from our study.

## Conclusion

Surgical management of patients with cingulate epilepsy is highly challenging, particularly when the MRI is negative. The localizing yield of non-invasive tests such as scalp EEG, PET, ictal SPECT and MEG in non-lesional cingulate epilepsy is relatively limited and ICEEG is often mandatory. MRI postprocessing could be incorporated into routine surgical evaluation to enhance detection of subtle epileptogenic abnormalities in this particularly challenging population.

## Ethics statement

This study was carried out in accordance with the recommendations of the institutional review board ethical guidelines of two hospitals (Cleveland Clinic Foundation and the Second Affiliated Hospital of Zhejiang University) with written informed consent from all subjects. All subjects gave written informed consent in accordance with the Declaration of Helsinki. The protocol was approved by the institutional review board ethics committee.

## Author contributions

ShaW contributed to the conception, design the study, analysis of the data, interpretation of the results, and drafting the manuscript. BJ revising the manuscript. TA analysis of the data. MK analysis of the data. SJ revising the manuscript. BK revising the manuscript. JG-M interpretation of the results. RP analysis the data. IN and AA interpretation of the results. ShuW, MD, and ZIW interpretation of the results, drafting the manuscript, and final approval of the version to be published.

### Conflict of interest statement

The authors declare that the research was conducted in the absence of any commercial or financial relationships that could be construed as a potential conflict of interest.

## Abbreviations

CE: cingulate epilepsy
CC: cingulate cortex
MAP: morphometric analysis program
FCD: focal cortical dysplasia.
